# Predicting miRNA-disease associations using a hybrid feature representation in the heterogeneous network

**DOI:** 10.1186/s12920-020-00783-0

**Published:** 2020-10-22

**Authors:** Minghui Liu, Jingyi Yang, Jiacheng Wang, Lei Deng

**Affiliations:** 1 School of Computer Science and Engineering,Central South University, Changsha, 410075 China; 2 School of Software, Xinjiang University, Urumqi, 830008 China

**Keywords:** miRNA-disease association, HeteSim measure, Diffusion feature, eXtreme gradient boosting

## Abstract

**Background:**

Studies have found that miRNAs play an important role in many biological activities involved in human diseases. Revealing the associations between miRNA and disease by biological experiments is time-consuming and expensive. The computational approaches provide a new alternative. However, because of the limited knowledge of the associations between miRNAs and diseases, it is difficult to support the prediction model effectively.

**Methods:**

In this work, we propose a model to predict miRNA-disease associations, MDAPCOM, in which protein information associated with miRNAs and diseases is introduced to build a global miRNA-protein-disease network. Subsequently, diffusion features and HeteSim features, extracted from the global network, are combined to train the prediction model by eXtreme Gradient Boosting (XGBoost).

**Results:**

The MDAPCOM model achieves AUC of 0.991 based on 10-fold cross-validation, which is significantly better than that of other two state-of-the-art methods RWRMDA and PRINCE. Furthermore, the model performs well on three unbalanced data sets.

**Conclusions:**

The results suggest that the information behind proteins associated with miRNAs and diseases is crucial to the prediction of the associations between miRNAs and diseases, and the hybrid feature representation in the heterogeneous network is very effective for improving predictive performance.

## Background

MicroRNAs(miRNAs) are a kind of small single-stranded endogenous non-coding RNAs with a length about 22 nucleotides, which play an important role in regulating the gene expression during the post-transcriptional level [1, 2]. Many studies have shown that the dysregulation of miRNAs is involved in multiple human diseases like cancers [3], cardiovascular diseases [4] and Alzheimer’s diseases [5] etc., and the prediction of miRNAs-diseases associations is crucial to understand the diseases pathogenesis [6]. Furthermore, George Adrian, et al. found that the miR15 and miR16 are deleted in a lot B cell chronic lymphocytic leukemias (B-CLL) [7], T. Sredni et al. demonstrated that miR-129 and miR-25 express abnormally in all pediatric brain tumor types [8]. Besides, Jun Lu et al. successfully classified poorly differentiated tumours using miRNA expression profiles [9], which demonstrated the potential of miRNAs as biomarkers. Therefore, Predicting miRNA-disease associations is very meaningful. However, a lot of miRNA-disease associations remain unknown and experimental approaches for predicting the associations are time-consuming and expensive. Therefore, a lot computational methods have been developed to predict the miRNA-disease associations.

Computational methods can be grouped into two categories: network-based methods and machine learning-based methods. Network-based methods usually use similarity measurement to predict the associations. For example, Jiang et al. [10] presented a computational method to predict the associations between miRNAs and diseases by prioritizing entire human microRNAome according to the disease of interest. The higher the rank is, the more possibly the miRNA can associate with the disease. In 2010, the model was improved by introducing genomic data [11]. However, the performance of the model was still not satisfactory because the known target genes of miRNAs are too rare to support the methods effectively. Chen et al. develpoed a method called RWRMDA [12], the author ran random walk with restart algorithm on a miRNA functional similarity network to obtain a score for every miRNA, and the miRNA with a higher score is more likely to associate with a certain disease. Shi et al. [13] extended random walk with restart algorithm (RWR), they used proteins associated with diseases and miRNAs as seed nodes to calculate the ES score by RWR respectively, and then used the *P*-value to predict whether the disease and miRNA are related. PRINCE [14] is another algorithm optimized based on RWR, it proposed a novel method to initial probability of miRNAs. However, these methods, based on RWR, are dependent on known associations between miRNAs and a given disease, so it couldn’t be applied to predict the relationships between miRNAs and a new disease, without any associations with miNRAs. Furthermore, defining a proper similarity calculation model is challengeable in this category.

The prediction models in another category are based on machine learning. For example, Xu et al. [15] extracted features from a miRNA-disease network, and then used the features to train a prediction model by support vector machine (SVM), the method can discover positive samples from massive negative samples. Chen et al. [16] presented a semi-supervised and global method RLSMDA, the method calculated possibilities of being associated with a given disease for each miRNA by a continuous classification function, and it could predict the associations of diseases and miRNAs without any known association between them. However, the method didn’t integrate the information related to miRNAs and diseases completely since the continuous classification function is established for the miRNA network and the disease network separately.

Recently, more computational methods are proposed. Zheng et al. [17] developed a machine leaning-based method MLMDA, which used a variety of information including miRNA sequence information, miRNA functional similarity, disease semantic similarity and Gaussian interaction profile kernel similarity information to train their model by applying random forest classifier. The classifier achieved promising performance, but it might take a lot of effort to prepare the required data. What’s more, the knowledge of deep learning was also applied in this field. Peng et al. [18] utilized a convolutional neural network to predict miRNA-disease association, input data was reduced miRNA-disease interaction features which were captured from a three-layer network. The similarity metric is essential in order to predict associations between miRNAs and diseases, where Yang et al. [19] used a novel method miRGOFS to measure functional similarities of miRNAs, and the method considered both common ancestors and descendants of GO terms when it was used to calculate similarities among GO sets in an asymmetric manner, so it can help predict the miRNA-disease associations. Chen et al. [20] presented the first decision tree, learning-based model, whose informative feature vectors were extracted from miRNA functional similarities, the disease semantic similarities, and known miRNA-disease associations. Yin et al. [21] put forward LWPCMF, they used weighted profile (WP), collaborative matrix factorization (CMF) and logistic function to optimize their model.

In this work, we present a computational method named MDAPCOM to predict the associations between miRNAs and diseases by combined features. First, we construct a miRNA-protein-disease global network by merging six subnetworks, which are miRNA-miRNA Similarity Network, Protein-Protein Interaction Network, Disease-Disease Similarity Network, miRNA-Target Interaction Network, miRNA-Disease Relationship Network and Protein-Disease Association Network respectively. Subsequently, we extract diffusion features for each node and a 39-dimensional HeteSim feature for each miRNA-disease pair in the global network. The diffusion features are extracted by random walk with restart algorithm and then reduced in dimension using the singular value decomposition algorithm (SVD). Finally, we integrate these two features to train the miRNA-disease association prediction model using eXtreme Gradient Boosting (XGBoost) algorithm. We apply the MDAPCOM method under 10-fold cross-validation and achieve an AUC of 0.991. MDAPCOM also performs better when compared with other two previous methods RWRMDA [12] and PRINCE [14], which also used network features for prediction. Furthermore, our method performs well on three unbalanced data sets with positive and negative samples ratios 1:2, 1:5 and 1:10, respectively.

## Results

### Data sources

We collect six different types of data from the Internet, which are the miRNA-miRNA similarity data, miRNA-Protein interactions, miRNA-Disease relationships, PPI data (Protein-Protein interactions), Protein-Disease association data, Disease-Disease similarity data, respectively, containing 2588 miRNAs, 18143 proteins and 5080 different kinds of diseases totally.

#### miRNA-miRNA similarity network

We obtain miRNA expression data from miRmine database [22]. In this database, the researchers analyzed overall expression profile of human, obtained from different miRNA-seq databases. It contains 2822 different precursor miRNAs where more than two of them consist one mature miRNA, so we can derive the expression values of every mature miRNA from the average values of its precursors’. In this way, we obtain 2588 miRNA expression profiles. Moreover, the Pearson Correlation Coefficient (PCC) scores are calculated to preform similarities of the expression profiles between two miRNAs [23]. The higher the PPC score is, the more likely these two miRNAs are similar. The miRNA-miRNA Similarity network is also built. In the network, every miRNA is a node and the PPC scores present the edges, and the negative edges are cut down.

#### Protein-protein interaction network

We derive data from the STRING database V10.0 [24]. The database offers data which is obtained from the results of biochemical experiments, biophysical or genetic techniques. We get 7,866,428 PPI entries from 18,143 proteins in the database and use them to build our Protein-Protein Interaction Network. In PPI network, each of the entry comprises a protein node A, a protein node B, and the predicted relationship’s score between them. The highest score means the two proteins can interact with each other with the biggest possibility and vice versa. Last, we utilize the predicted score to present the value of each edge between two protein nodes to construct our Protein-Protein Interaction Network.

#### Disease-disease similarity network

To build the Disease-Disease Similarity Network, we obtain data from the MimMine database. [25] It is mapped from OMIM database, containing more than 5000 human genetic disease phenotypes. It is worthy to point out that we normalize disease-disease similarities’ values into [0,1] in MimMiner database. Subsequently, we receive 5080 kinds of diseases and get the similarities between them. Finally, we construct the Disease-Disease Similarity Network where each node presents a kind of disease, and the weight is similarity between them.

#### miRNA-target interactions network

We download miRNA-target interactions from the miRTarBase database of release 7.0 [26], miRNA-Target Interaction Network can be built. It should be point out all data is validated in this database. Moreover, we map the genes onto protein entries, and remove invalid entries (miRNA or protein), which are repeated and out-of-range. Finally, we extract miRNAtarget interactions between 2,588 miRNAs and 18,143 proteins. Then, miRNA-Target Interaction network is constructed based on these data.

#### miRNA-disease relationship network

We get miRNA-disease data from HMDD v3.0 database [27], which is a reliable online database containing 1102 gene on miRNA, 850 different types of diseases and 32281 associations between miRNA and disease, and they are all based on literature. Furthermore, we receive the relationships between 2588 miRNAs and 5080 diseases which are mentioned above. Lastly, we build the miRNA-Disease Relationship network using these validated data.

#### Protein-disease association network

We obtain data from DisGeNET database [25] which collects data on genotype-phenotype relationships. In this work, we map genes into proteins and unify the name of diseases, so 18,143 proteins,5080 diseases and the associations between them are extracted. Then, we construct a Protein-Disease Association Network from these data.

#### Global heterogeneous network

We integrate the aforementioned networks to build the global heterogeneous network:

$$T= \left[\begin{array}{ccc} M& B& C\\ B^{T}& P& W\\ C^{T}& W^{T}& D\\ \end{array}\right] $$

where T represents our global heterogeneous network, M, P, D present similarity of miRNA-miRNA, protein-protein and disease-disease respectively, B presents the miRNA-Target Interaction Network, C indicates miRNA-Disease Relationship Network, and W shows the Protein-Disease Association Network. Obviously, the *B*^*T*^,*C*^*T*^ and *W*^*T*^ are transposed matrices of B, C and W, and the edges with value less than 0.5 are removed from the network.

There are 2588 miRNAs and 5080 diseases in our miRNA-protein-disease global network, so we can get a total of 13147040 (2588 ×5080) miRNA-disease pairs. We extract a 639-dimensional combined feature vector for each miNRA-disease pair in the global network, in which 11824 feature vectors are positive samples while the other 13135216 feature vectors are negative samples. We randomly select 11824 feature vectors from 13135216 negative samples to construct a standard dataset together with 11824 positive samples, subsequently, we execute 10-fold cross-validation on the standard dataset. The positive and negative samples are randomly divided into 10 subsamples equalled in size(the size of the tenth subsample is 1186 because 11824 is’t divisible by 10), one of which is retained as the validation set and the other 9 subsamples are regarded as the training set. Then the procedure iterates 10 times with each one in the 10 subsamples as the validation set, before each iteration, the associations occurred in the validation set are removed from the original global network, and then all feature vectors are re-extracted from the new global network. Furthermore, another three unbalanced data sets are obtained in the same way except the size of the selected negative samples, and the size of negative samples in three unbalanced data sets is 23648, 59120 and 118240, respectively.

### Performance measures

We apply 10-fold cross-validation, and obtain the average performance of our model through the performance evaluation. In terms of performance evaluation, we select precision(PRE), recall(REC), F-score(FSC), accuracy(ACC) and the area under the receiver operating characteristic curve(AUC):

$$\begin{array}{@{}rcl@{}} &&PRE = \frac{TP}{TP+FP},\\ &&REC = \frac{TP}{TP+FN},\\ &&ACC = \frac{TP+TN}{TP+TN+FP+FN},\\ &&FSC = \frac{2\times PRE\times REC}{PRE+REC}, \end{array} $$

TP and FP are the amount of correctly predicted positive and negative samples, FP and FN are the numbers of positive and negative samples predicted by mistakes. Simultaneously, we calculate the area under ROC curve (AUC) to measure the overall performance.

### Excellent combined feature

**Fig. 1:**
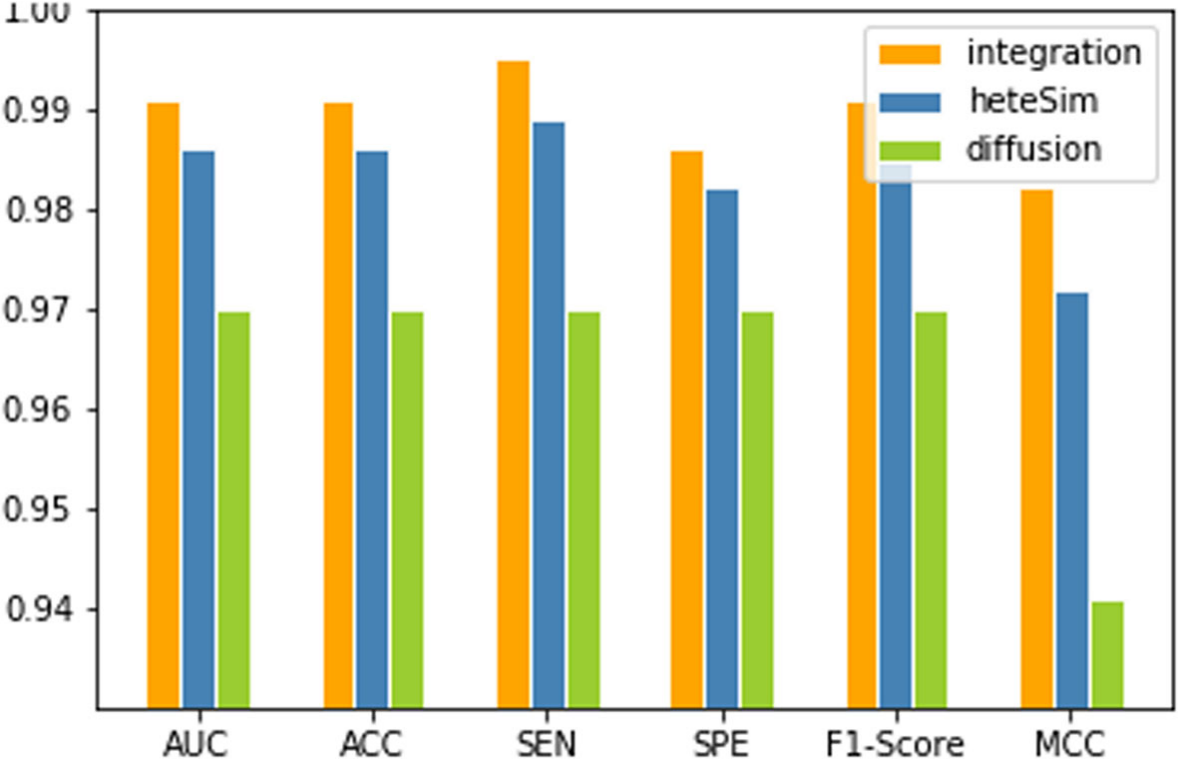
Performance comparison of different feature groups (Diffusion, HeteSim and combined feature)

In our method, we extract two different features from a global heterogeneous network, a global matrix of nine different data, and combine them to construct our training dataset. Firstly, with the help of random walk with restart algorithm, we extract diffusion feature of each node from our global network, so we can get a 20588*20588 feature matrix, where a row represents a feature vector of one node. For example, the first row shows the miRNA1’s feature vector, the 2589 th row is the protein1’s feature vector, and the 20732 th row is the disease1’s feature vector. In the next step, we apply SVD algorithm on this feature matrix to reduce the dimension of it from 20588 to 300, here our feature matrix is 20588*300. After obtaining reduced feature vectors of each node, we combine each miRNA’s feature vector with each disease’s, so we get a (2588*5080) * 600 miRNA-disease feature matrix, where a row shows the feature vector of a pair of miRNA-disease. Secondly, we calculate HeteSim scores of each miRNA-disease pair, and get a (2588*5080) * 39 HeteSim matrix. Finally, in order to construct our training data, we joint the SVD feature and HeteSim score, so we get a (2588*5080) * 639 feature vector, where a row is the combined feature vector of a miRNA-disease pair. To show excellent performance of our method, we use diffusion features, the HeteSim feature and the combined feature to train the prediction model using 10-fold cross-validation under the standard data set, respectively, and the result shows in Fig. 1. The AUC value of training model with the diffusion feature and the HeteSim feature reach 0.970 and 0.986, respectively, and we get an AUC of 0.991 using combined feature.

### Superiority of XGBoost algorithm

**Fig. 2:**
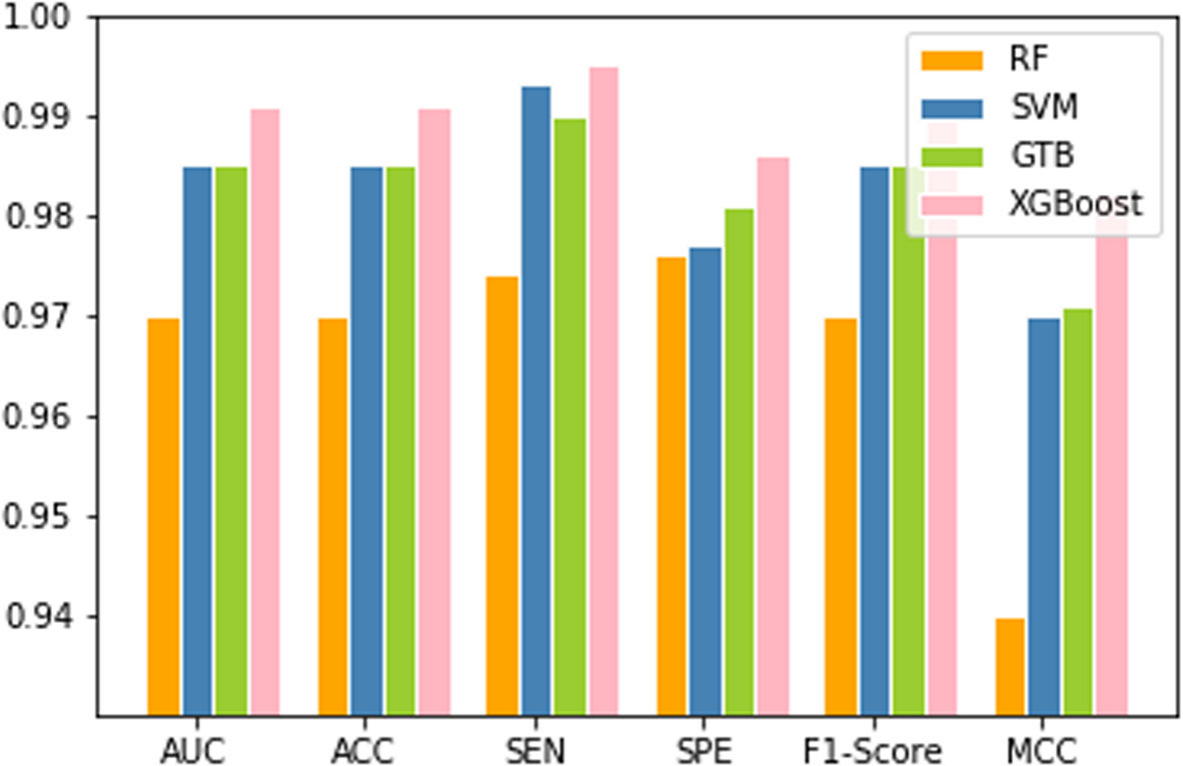
Comparison result between XGBoost and other machine learning algorithms including PF, SVM and GTB

In this work, we apply eXtreme Gradient Boosting(XGBoost) [28] algorithm to train our model. We compare XGBoost algorithm with other machine learning algorithm to present that the eXtreme Gradient Boosting(XGBoost) algorithm is the most suitable method for us. To achieve the goal, we obtain other classifiers from python toolkits scikit-learn and apply 10-fold cross-validation. We compare XGBoost algorithm with random forest (RF) [29], support vector machine (SVM) [30]and gradient tree boosting (GTB) [31] algorithm. In random forest algorithm, we set the minimize samples split to 42, maximize depth of tree to 11 and the resting parameter values to default. In the support vector machine algorithm, we use RBF kernel setting the C value to 100, gamma value to 0.0001. In gradient tree boosting algorithm, we set the minimize samples split to 110, the maximize depth of tree to 9. The results perform in Fig. 2.

### Performance comparison with existing methods

**Fig. 3:**
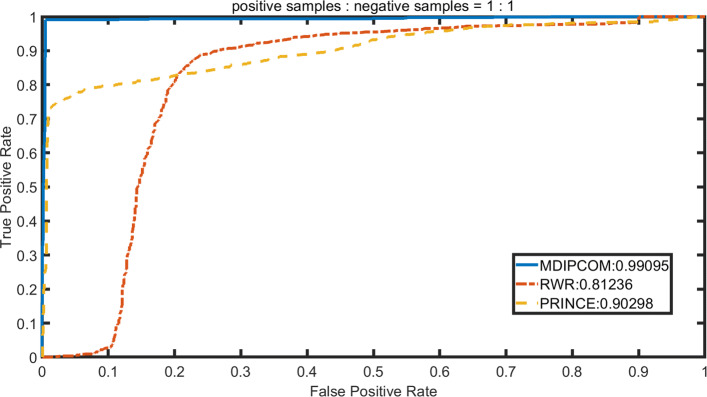
The ROC curves of MDAPCOM and previous approaches containing PRINCE and RWRMDA on the data sets. The ratio of positive and negative sample 1:1

**Fig. 4:**
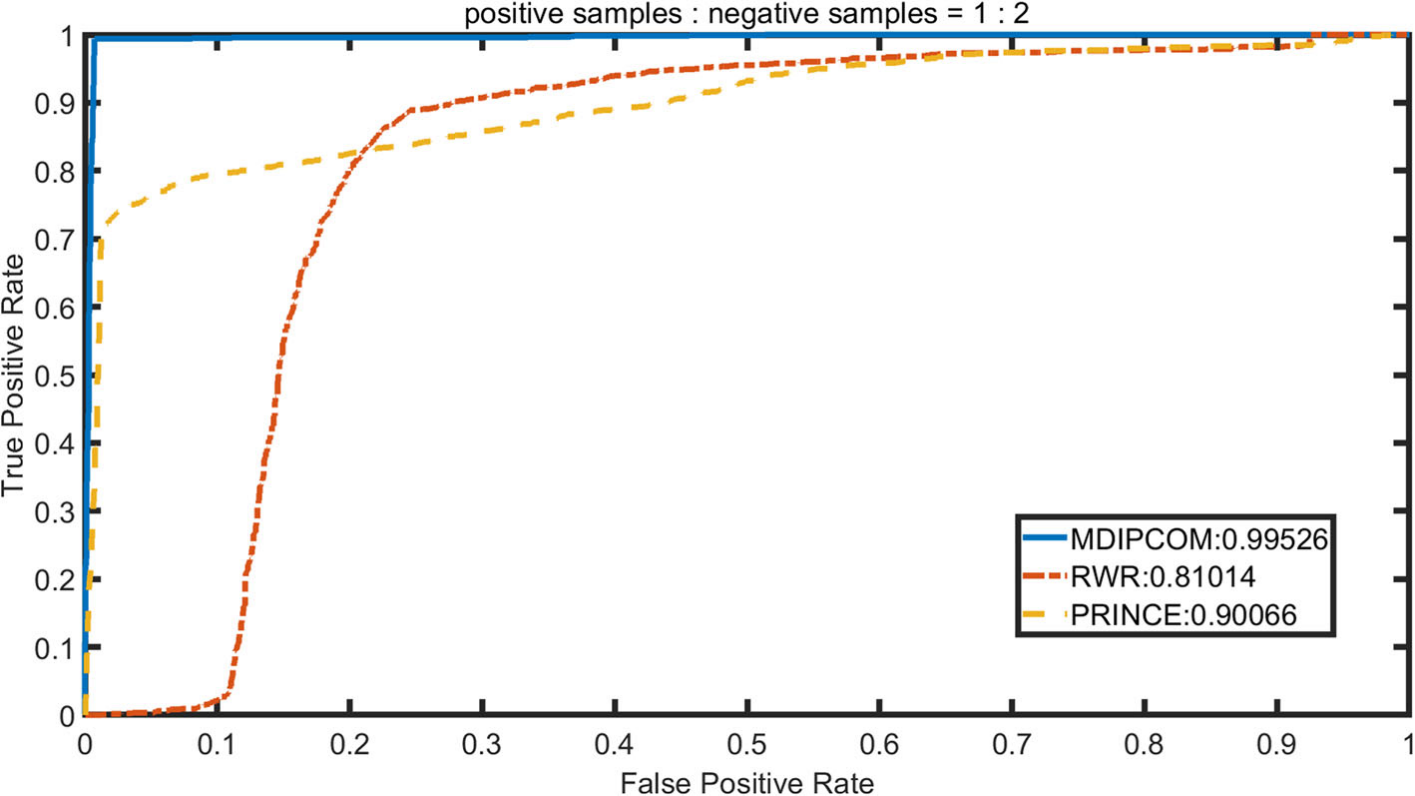
The ROC curves of MDAPCOM and previous approaches containing PRINCE and RWRMDA on the data sets. The ratio of positive and negative sample 1:2

**Fig. 5:**
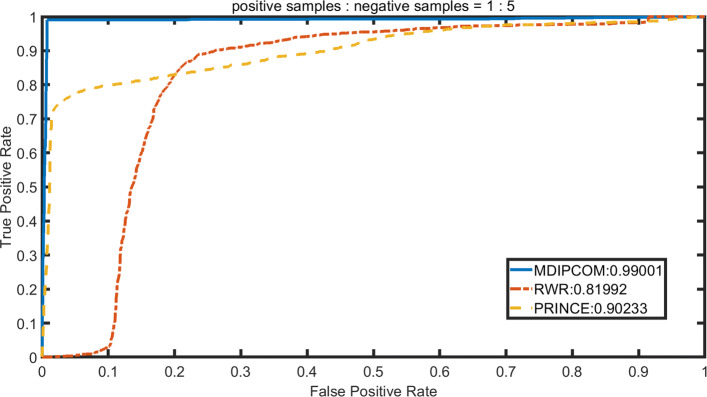
The ROC curves of MDAPCOM and previous approaches containing PRINCE and RWRMDA on the data sets. The ratio of positive and negative sample 1:5

**Fig. 6:**
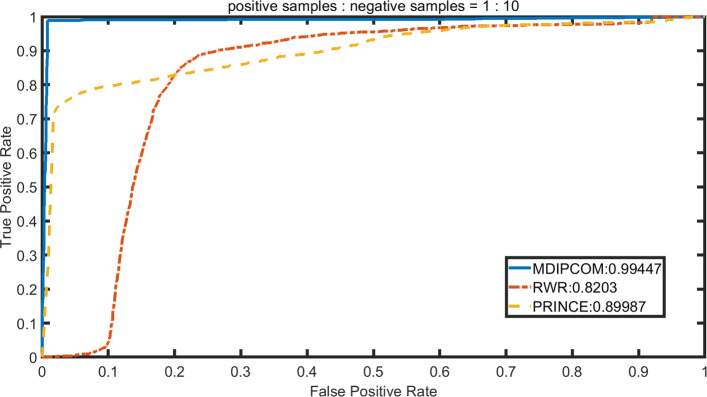
The ROC curves of MDAPCOM and previous approaches containing PRINCE and RWRMDA on the data sets. The ratio of positive and negative sample 1:10

We implement RWRMDA [12] and PRINCE [14] under a standard dataset and three unbalanced datasets, applying 10-fold cross-validation to calculate their AUC values and compare theirs with MDAPCOM’s. For PRINCE, we set *α*=0.95, d=log (9999), c=-15 and then apply the random walk with restart 10 times. The probability of restarting in RWRMDA is set to 0.5. To visually describe and compare the performance of the three methods, we plot the Receiver Operating Characteristic (ROC) curve with its horizontal axis representing false positive rate (FPR) and the vertical axis representing true positive rate (TPR). Subsequently, we use the area under the ROC curve (AUC) to accurately compare the performance of the three methods. Figures 3, 4, 5 and 6 show the performance of the three methods under four datasets with different positive and negative ratios, respectively. Among three methods, MDAPCOM significantly outperforms the other two methods, achieving an amazing AUC score 0.99. Furthermore, the AUC scores of our method are all above 0.99 under four data sets, which proves its stability.

## Conclusions

In this work, we present a prediction method based on machine learning to predict the associations between miRNAs and diseases, MDAPCOM. We build a miRNA-protein-disease global network, then extract dimensional reduced RWR diffusion feature and HeteSim feature from the network, the diffusion feature reflects the node topological information in the heterogeneous network and the HeteSim feature extracts the correlation of node pairs. Subsequently, the two features are combined to train the miRNA-disease association prediction model using 10-fold cross-validation by eXtreme Gradient Boosting (XGBoost). The MDAPCOM shows better performance than other two previous methods, based on network feature. The excellent performance suggests that the information behind proteins which are associated with miNRAs and diseases is crucial to predict associations between miRNAs and diseases. Furthermore, the two features extract network information from different perspectives and the combination of them integrates network information effectively, which also contributes to the excellent performance of the method.

## Methods

### Overview of MDAPCOM

**Fig. 7:**
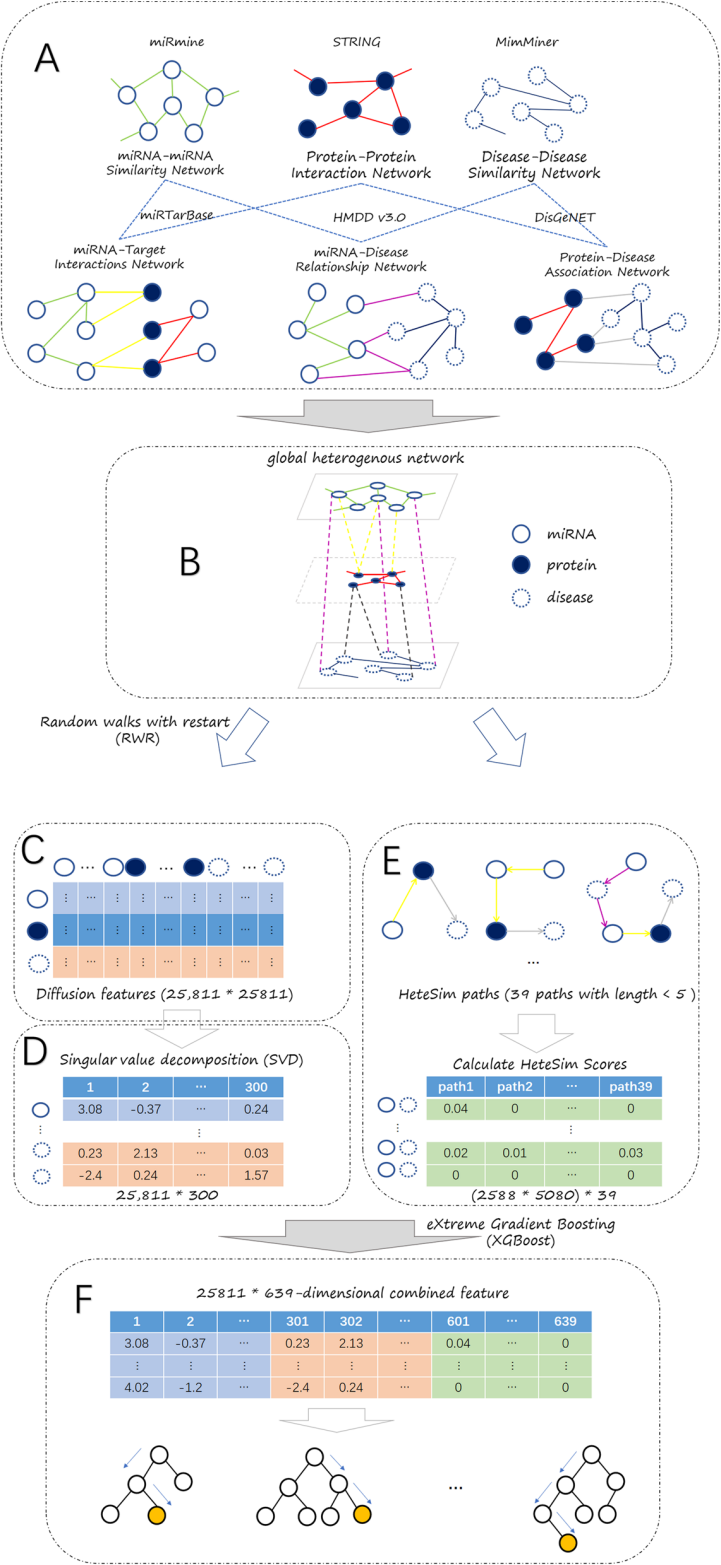
The flowchart of MDAPCOM: **a** Obtain six kinds of data from online databases. **b** Merge these data to build a global heterogeneous network **c** Utilize RWR algorithm to get the diffusion feature. **d** Apply SVD to reduce dimension of the diffusion feature. **e** Use HeteSim measure to obtain HeteSim feature. **f** Integrate reduced diffusion feature and HeteSim feature and then apply XGBoost algorithm to train the model using the combined feature

Our method is displayed in Fig. 7, which is built through following steps: (A) Collect six types of data sources and remove invalid and repeated data. (B) Merge the six networks to build a global miRNA-protein-disease heterogeneous network. (C) Run random walks with restart (RWR) algorithm in the global network to calculate a diffusion feature for every node, which reflects the relevance of one node with all other nodes (miRNAs, proteins and diseases) in the network (D) Run the singular value decomposition (SVD) algorithm to reduce dimension of the diffusion feature, obtaining a 300-dimensional feature vector for every node. (E) Use HeteSim measure to estimate the correlation between two nodes and get a 39-dimensional HeteSim feature for each miRNA-disease pair. (F) Integrate the 600-dimension diffusion feature(300-dimensional for miRNA and 300-dimensional for disease) and 39-dimensional HeteSim feature to train a miRNA-disease association prediction model by eXtreme Gradient Boosting (XGBoost).

### Diffusion feature of reduced dimension

To predict the miRNA-disease associations, we transform the problem to obtain possibility that a miRNA can associate with a disease. The Random Walk with Restart algorithm can capture the relationships between two nodes and the global topological information of nodes in the network [32–34]. In this study, we run RWR algorithm on the global heterogeneous network and get a high-dimensional(25,811) vector for each node. The vector reveals the topological properties of the node in the network, which includes a set of possibilities that a node can access to other nodes. We use D to represent the adjacency matrix of our global heterogeneous network, and T, a normalized matrix, represents the transition probability from the node i to the node j, T is defined as

1 $$ T_{ij} = \frac{D_{ij}}{{\sum\nolimits}_{k}D_{ik}}  $$

If a node i is connected with a node j, the value of *D*_*ij*_ is 1, otherwise the value is 0. The RWR can be regarded as an iterative process, which is expressed as

2 $$ P_{t+1} = (1-\alpha)TP_{t}+\alpha P_{0}  $$

Where *α* is the restart rate of random walker which is in the range of [0,1], *P*_0_ is the initial probability of the heterogeneous network, *P*_*t*_ is the state of the heterogeneous network when the process is in the t-th.

Here, we get a 25,811-dimensional feature for every node which reveals the topological relevance of a node to other nodes(2,588 miRNAs, 18,143 proteins and 5,080 diseases) in the network. Using such tremendous features directly to train the model is pretty time-consuming and unnecessary, since they contain some noise. Therefore, we reduce the 25,811-dimensional diffusion feature to 300-dimension by singular value decomposition (SVD) algorithm [35, 36].

### HeteSim measure

The HeteSim measure performs well in measuring the correlation of nodes in the heterogeneous biological network [37]. It’s a self-maximum and symmetric measure, using an uniform framework to measure the correlation of nodes based on specified path [38]. In this paper, we use HeteSim scores of miNRA-disease pairs to extract network information.

#### **Definition 1**

(**Transition probability matrix** [38]) A and B are two types of nodes in the heterogeneous network. (*M*_*AB*_)_*m*∗*n*_ is an adjacency matrix indicating the relation between A and B, if there is an association between a node i belonging to A and a node j belonging to B, *M*_*AB*_(*i*,*j*)=1, otherwise *M*_*AB*_(*i*,*j*)=0. The transition probability matrix *T*_*AB*_ is defined as follows

3 $$ T_{AB}(x, y) = \frac{M_{AB}(x, y)}{{\sum\nolimits}_{i = 1}^{n}M_{AB}(x, i)}  $$

#### **Definition 2**

(**Reachable probability matrix** [38]) *R*_*ρ*_ represents the reachable probability matrix based on the path $ \rho = P_{1}P_{2}P_{3} \dots P_{n + 1} $, where *P*_*i*_ represents any types of nodes of the heterogeneous network. *R*_*ρ*_ can be calculated as

4 $$ R_{\rho} = T_{P_{1}P_{2}}T_{P_{2}P_{3}} \dots T_{P_{n}P_{n + 1}}  $$

Based on the above 2 definitions, we can calculate the HeteSim score in 3 steps [38].

1. Separate the path *ρ* from the middle into *ρ*_*L*_ and *ρ*_*R*_. When the path length is even, *ρ*_*L*_ and *ρ*_*R*_ are equal in length, and $ R_{\rho _{L}} $ and $ R_{\rho _{R}} $ can be directly calculated. When the path length is odd, there are two intermediate nodes, take each one of them as intermediate node respectively to obtain $ \rho _{L_{1}} $, $ \rho _{L_{2}} $, $ \rho _{R_{1}} $ and $ \rho _{R_{2}} $, then $ R_{\rho _{L}} $, $ R_{\rho _{R}} $ can be calculated as
  $$R_{\rho_{L}} = \frac{R_{\rho_{L_{1}}} + R_{\rho_{L_{2}}}}{2} $$
  $$R_{\rho_{R}} = \frac{R_{\rho_{R_{1}}} + R_{\rho_{R_{2}}}}{2} $$
2. Calculate the $ R_{\rho _{_{L}}} $ and $ R_{\rho _{R}^{-1}} $, where $ \rho _{R}^{-1} $ represents the reverse of *ρ*_*R*_, for example, if *ρ*_*R*_=*ABC*, then $ \rho _{R}^{-1} = CBA $.
3. Achieve the HeteSim measure as
  5 $$ HeteSim\left(a,b|\rho\right) = \frac{ R_{\rho_{L}}(a, :)\left(R_{\rho_{R}^{-1}}(b,:)\right)^{T} } { {\lVert R_{\rho_{L}}(a, :) \rVert}_{2} \times {\lVert R_{\rho_{R}^{-1}}(b,:) \rVert}_{2} }  $$

Using the above method, we can derive 39 HeteSim scores for each miRNA-disease pair(i.e. a 39-dimensional HeteSim feature vector for each miRNA-disease pair) based on all paths less than 5 in length starting at miRNA and ending at disease. The detailed paths are listed in Table 1.

**Table 1:** All paths less than 5 in length starting at miRNA and ending at disease. M is miRNA, P is protein and D is disease, for example, path1 MMD is the path miRNA-miRNA-disease

id	path	id	path	id	path
1	MMD	14	MMMPD	27	MPPDD
2	MPD	15	MMMDD	28	MPDMD
3	MDD	16	MMPMD	29	MPDPD
4	MMMD	17	MMPPD	30	MPDDD
5	MMPD	18	MMPDD	31	MDMMD
6	MMDD	19	MMDMD	32	MDMPD
7	MPMD	20	MMDPD	33	MDMDD
8	MPPD	21	MMDDD	34	MDPMD
9	MPDD	22	MPMMD	35	MDPPD
10	MDMD	23	MPMPD	36	MDPDD
11	MDPD	24	MPMDD	37	MDDMD
12	MDDD	25	MPPMD	38	MDDPD
13	MMMMD	26	MPPPD	39	MDDDD

### The eXtreme gradient boosting (XGBoost) algorithm

The eXtreme Gradient Boosting is an end-to-end system extended by tree boosting, and it’s used widely in machine learning [28]. The algorithm can be obtained from python toolkits scikit-learn. In this study, a 600-dimensional diffusion feature(300-dimensional for miRNA and 300-dimensional for disease) and a 39-dimensional HeteSim feature are extracted for each miNRA-disease pair in the global network. Subsequently, the two features are combined, forming a 639-dimensional feature, to train the prediction model by XGBoost, where the optimal learning rate is 0.15, the number of iterations is 650, the max depth of tree is 4 and default values set for the other parameters.

## Abbreviations

XGBoost: eXtreme Gradient Boosting
miRNA: MicroRNA
RWR: random walk with restart algorithm
SVM: support vector machine
GO: gene ontology
WP: weighted profile
CMF: collaborative matrix factorization
SVD: singular value decomposition
PPI: Protein-Protein interaction
PCC: Pearson correlation coefficient
ROC: receiver operating characteristic curve
AUC: area under the receiver operating characteristic curve
PRE: precision
REC: recall
FSC: F-score
ACC: accuracy
RF: random forest
GTB: gradient tree boosting
FPR: false positive rate
TPR: true positive rate
RBF: Radial Basis Function
